# HIV-1 group O integrase displays lower susceptibility to raltegravir and has a different mutational pathway for resistance than HIV-1 group M

**DOI:** 10.7448/IAS.17.4.19738

**Published:** 2014-11-02

**Authors:** Agnès Depatureaux, Thibault Mesplède, Peter Quashie, Maureen Oliveira, Daniela Moisi, Bluma Brenner, Mark Wainberg

**Affiliations:** McGill AIDS Center, Lady Davis Institute for Medical Research, Jewish General Hospital, Montreal, Canada

## Abstract

**Introduction:**

HIV-1 group O (HIV-O) is a rare HIV-1 variant characterized by a high number of polymorphisms, especially in the integrase gene, e.g. positions L74I, S153A, G163Q and T206S. As HIV-O integrase enzymes have not previously been studied, our aim was to assess the impact of HIV-O integrase polymorphisms on susceptibility to integrase inhibitors and emergence of resistance associated mutations.

**Viruses and Methods:**

We cloned and purified integrase proteins from each of HIV-1 Group O clades A (HIV-O/A) and B (HIV-O/B), a HIV-O divergent strain (HIV-O/Div), and HIV-1 group M (subtype B, HIV-M/B) and characterized these enzymes for susceptibility to integrase strand transfer inhibitors (INSTIs) in cell-free assays and in tissue culture, in the absence or presence of varying concentrations of several INSTIs. The inhibition constant (Ki) and IC50 were calculated and compared for HIV-M and HIV-O integrases. Selections for resistance-related mutations were performed using cord blood mononuclear cells and increasing concentration of INSTIs.

**Results:**

HIV-O integrase and viruses were more susceptible to raltegravir (RAL) in competitive inhibition assays and in tissue culture than were HIV-M enzymes and viruses, respectively. During selection, we observed different pathways of resistance depending on the drug and clade. Mutations selected in HIV-O can be classified as follows: (1) mutations described for HIV-M such as T97A, Q148R, V151A/I (RAL), T66I, E92Q, E157Q (EVG) and M50I, R263K (DTG) and (2) signature mutations for HIV-O (i.e. not described in HIV-M) F121C (HIV-O/B for RAL), V75I (HIV-O/A for RAL) and S153V (HIV-O/A for DTG). Only the HIV-O/Div selected the Q148R mutation for RAL and R263K+M50I for DTG, as previously described for HIV-M. None of the HIV-O viruses selected either N155H or Y143C. The selection of the specific S153V mutation could be explained at the nucleotide level: HIV-O at this position contains an alanine and substitution of alanine to valine (153AGGC→153VGTC) is easier than substitution of alanine to tyrosine (153AGGC→153YTAC), with only a transversion needed instead of one transition plus one transversion.

**Conclusions:**

This is the first report of susceptibility and resistance in vitro to INSTIs for HIV-O. Our study confirmed the impact of HIV-O polymorphism, on susceptibility to INSTIs and the emergence of resistance mutations.

